# Farm Environmental Enrichments Improve the Welfare of Layer Chicks and Pullets: A Comprehensive Review

**DOI:** 10.3390/ani12192610

**Published:** 2022-09-29

**Authors:** Dan Xu, Gang Shu, Yanting Liu, Pingwu Qin, Yilei Zheng, Yaofu Tian, Xiaoling Zhao, Xiaohui Du

**Affiliations:** 1 Key Laboratory of Livestock and Poultry Multi-omics (Ministry of Agriculture and Rural Affairs), Institute of Animal Genetics and Breeding, College of Animal Science and Technology, Sichuan Agricultural University, Chengdu 611130, China; 2 Farm Animal Genetic Resources Exploration and Innovation Key Laboratory of Sichuan Province, Sichuan Agricultural University, Chengdu 611130, China; 3 Department of Basic Veterinary Medicine, Sichuan Agricultural University, Chengdu 611100, China; 4 College of Veterinary Medicine, University of Minnesota, Minneapolis, MN 55108, USA

**Keywords:** farm environmental enrichment, welfare, layer chicks and pullets, manipulable material, structural equipment

## Abstract

**Simple Summary:**

Layer industries that are still using cage housing systems should encourage the provision of appropriate farm enrichments, especially at an early stage. Farm environmental enrichments such as litter, sand, alfalfa bales, chick papers, pecking stones, pecking strings, perches, slopes, elevated platforms, aviaries and outdoor access reduce feather and skin damage, as well as abdominal fat content. Additionally, they promote the development of the brain and musculoskeletal systems and improve biological function, productivity and product quality with lifelong benefits for hens by continuously exposing them to free access to forage, dust baths and free locomotion. In this review, we summarize several common and effective methods of farm environmental enrichments, including different manipulable materials, structural equipment, and outdoor access, moreover, the competence of the farm staff is a requirement to achieve useful utilization of these farm environmental enrichments that aim to reduce stress and improve the welfare and productivity of layers. Proper farm environmental enrichments benefit the wellbeing of caged birds.

**Abstract:**

Currently, cage housing is regarded as a global mainstream production system for laying hens. However, limited living space and confinement of birds in cages cause welfare and health problems, such as feather pecking, osteoporosis, obesity, and premature aging. Many studies have been conducted to alleviate layer welfare problems by providing farm environmental enrichments such as litter, sand, alfalfa bales, chick papers, pecking stones, pecking strings, perches, slopes, elevated platforms, aviaries and outdoor access with a trend towards complex enrichments. The provision of appropriate enrichments continuously attracts layers towards pecking, foraging, dust bathing, and locomotion, thereby giving lifelong benefits to laying hens. Hence, raising chicks and pullets under such conditions may reduce feather and skin damage, as well as accumulation of abdominal fat, and improve several biological features such as health, productivity, quality products, and docility of laying hens. Therefore, providing enrichment during the first few days of the layer’s life without any interruption is crucial. In addition, due to different farm conditions, environmental enrichment should be managed by well-trained farm staff. For example, in preventing feather pecking among the birds, litter materials for foraging are superior to dust bath materials or new items. However, a limited supply of litter creates competition and challenges among birds. Therefore, providing farm environmental enrichment for layers requires proper handling, especially in commercial layer farms. Hence, improving the welfare of chicks and pullets through optimizing on-farm environmental enrichments is essential for production systems practicing cage housing.

## 1. Introduction

Chickens are gregarious animals that interact closely within the flock by engaging in daily activities such as foraging, ground scratching, dust bathing, and preening. However, confining laying hens in narrow battery cages prevents them from exhibiting these natural behaviors that are beneficial to them, thereby causing chronic stress, poor health, pecking behavior, osteoporosis, obesity, and making layer chicks vulnerable to other diseases [1,2].

During the developmental stages, layer chicks start to peck, adapt to appropriate feed substrates, perch during the first few days of life, and develop fear-related resistance to unfamiliar objects [3,4,5]. Furthermore, studies have shown that layer chicks exposed to stress conditions during the rearing period suffer short-term, long-term, and transgenerational negative effects [6], thereby underpinning the importance of farm modifications to prepare birds for an optimal laying cycle.

Therefore, the aim of this review is to summarize the common and effective methods of farm environmental enrichments to reduce stress and improve welfare and productivity of layer chicks. These enrichments include different manipulable materials, structural equipment and outdoor access [6,7]. Furthermore, providing farmers with skills and technical know-how on the management of environmental enrichments is important, because they are responsible for ensuring the welfare of the chickens [7]. Since most countries still practice cage housing systems, the most effective and economical strategy to improve the welfare of layers is to adopt farm environmental enrichment during the rearing period [7].

## 2. Timing and Deep Understanding of Farm Environmental Enrichment in Layer Chicks and Pullet Production Systems

In this review, we selected common, available and accessible farm environmental enrichments with generally positive effects, not excluding a few with neutral or negative impact. Among the selected farm environmental enrichments, we strongly believe that the earlier the enrichment, the better the welfare objectives that will be achieved. Therefore, we firstly discuss the timing and interpretation of the enrichment.

### 2.1. Impact of Timing on Environmental Enrichment Layer Chicks and Pullets

Studies have shown that provision of litters or other enrichment materials for pullets and layers could reduce the frequency of feather pecking which could be effective through adulthood [5,8,9,10,11,12,13]. In addition, it exerts a positive effect on egg weight, total egg mass, and feed conversion ratio, and reduces mortality among the birds during the laying period [14].

Other studies have reported that chicks start foraging, sand bathing, and using perches during the first few days of life and develop fear-related resistance to unfamiliar objects [3,4,5,15]. Furthermore, early exposure to varied stimulation reduces fear [16] and feather pecking among the birds [17], and also increases the development of navigation skills of birds [18]. Birds are sensitive to environmental factors during the initial period of growth and development. Thus, providing chicks with access to early appropriate furnishings and enrichment materials, especially during the first few days of life, may have long-lasting effects, thereby reducing the risk of abnormal behavioral development. For instance, 10-day-old birds exposed to sand or litter show severe feather pecking and a higher mortality rate than those with free access to sand from day one [8,14,19]. Similarly, 2-week-old pullets raised in the aviary exposed to litter such as sawdust or straw also exhibited severe feather pecking and feather damage at 5 and 14 weeks old compared to those exposed to litter from birth [9]. Another study suggested that depriving birds of free access to simple litter materials such as chick paper with feed in the first four weeks contributed to severe feather pecking at five weeks old and could induce feather damage and fear during the entire rearing period [20]. Thus, early exposure to a complex rearing environment may improve the welfare of birds including enhancing appropriate behavior, and body and mental health, thereby easing the transition to the layer system, as well as reducing feather pecking and cannibalism through the reduction of fear, stress, emaciation, and dehydration. Therefore, providing appropriate farm environmental enrichment for layers at an early stage is beneficial. Thus, provision is recommended.

### 2.2. Further Understanding of Farm Environmental Enrichment in Animals

Studies involving human, rat, and mice models reported that the underlying mechanism through which early environmental enrichments could directly impact the birds’ welfare and health. Studies have shown that early life stress (ELS) negatively affects neurobehavioral relationships, and also induces cognitive impairment in later life by regulating the development of the hypothalamic pituitary adrenal axis, and changing the intestinal flora that leads to certain neurological disorders, including anxiety or depression [21,22,23].

However, farm environmental enrichments may effectively alleviate visceral and body hypersensitivity caused by early stress through neuro-regulation including regulation of neurogenesis, development of dendrites, and expression of neurotrophic growth factor. These effectively decrease the incidence of depression, anxiety, and stress [24]. Based on these results, it is easier to understand the beneficial effects of farm environmental enrichments on the welfare of layers and their possible mechanisms. Jeyaraj et al. (2021) reported that farm environmental enrichments enhance brain development and other biological functions and the general performance of birds. In addition, farm environmental enrichments reduce chronic stress by regulating the function of the brain–gut axis in layers, especially during the early stages [24]. Gut microbiotas have positive effects on integrating fearfulness, plasma corticosterone, and gene expression in the hypothalamus. Once chickens are in a state of stress, the secretion of hormones and neuropeptides in the gut ultimately invokes the release of cortisol from the adrenal gland via signals through the hypothalamus [25]. Birds provided with perches and litter materials show a decrease in plasma corticosterone and fearfulness, accompanied by decreased relative mRNA expression of glucocorticoid receptor (GR) and increased mRNA expressions of stress-related genes such as corticosterone-releasing hormone (CRH), brain-derived neurotrophic factor (BDNF), and N-methyl-d-aspartic acid receptor subunit 2A (NR2A) in the hypothalamus after a predator test [25]. In addition, there is a significant change in the composition and function of the intestinal microbiota [26]. Farm environmental enrichments also improve the function of the brain–gut axis and the development of skeletal muscle and nerves [26]. The improvement of the mechanisms of the brain–gut axis may exert long-term beneficial effects on daily behavior, reduce fear and pecking [26], enhance hatchability and viability [27,28,29], as well as improve egg quality parameters [30]. Therefore, many countries have advocated for farm environmental enrichments to improve biological function and general performance of layers, thereby promoting poultry welfare [31,32].

## 3. Effects of Manipulable Materials

Feather and aggressive pecking cause feather and skin damage. Exploratory pecking directed at the ground or at substrate, in contrast, does not cause feather and skin damage. The severity of feather pecking can be divided into two levels. Gentle feather pecking is exploratory or a stereotypic behavior [33] and does not generally develop into severe feather pecking [34,35]. However, severe feather pecking, by which layers pluck feathers from their companions for fiber rather than simply swallowing naturally shed feathers is common in chicks reared at high density and causes pain and feather damage, resulting in low egg production and a high mortality rate [36].

Studies have shown that the absence of foraging materials on the ground contributes to feather pecking behavior [37], especially for densely reared layers with untrimmed beaks [38]. However, this does not mean that an increase in foraging behavior will completely eradicate feather pecking behavior. For example, active pullets exhibit a high level of foraging behavior and are more likely to peck at the lower-status individuals with novel feather color and damaged or wrinkled feathers [39]. Thus, it is recommended to isolate low-status layers from the flock.

Studies have reported that foraging behavior in juvenile birds at 3–15 weeks old is positively correlated with feather pecking in adult birds at 17–37 weeks old [35,40], and other studies have reported the coefficient of 0.41 [41,42]. Therefore, aggressive behavior induced by mixing flocks such as feather color and body sizes mainly contributes to head and neck lesions, whereas the feather damage at the back part of the body is usually caused by pecking addiction [41,43]. Therefore, providing manipulable materials for layers as enrichments alleviates pecking. Hence, the importance of selecting suitable enrichment materials is undisputable.

### 3.1. Litter Materials for Foraging and Dustbathing

Several studies have tested the suitability of available litter materials. Table 1 shows that the provision of appropriate litter materials and litter quality is important for promoting foraging, dustbathing, and reducing pecking among layer chicks and pullets. Pullets are selective in terms of foraging material, depending on their physiological behaviors, as well as their behavior with their peers. Generally, birds prefer materials such as peat, sand, and wood chips that easily enter their feathers for dustbathing, and long straw for foraging [19,44,45]. Studies have reported that in cage rearing, the provision of chick paper from day one reduces feather damage and fear among the birds at 30 weeks old [12,46]. Various manipulable substrates such as sand, wood shavings, long-cut straw, chopped straw, polystyrene blocks, or polyester beads that were offered to flocks in the first few weeks influenced their choice later in life [8]. However, the impact of these materials was significant, and the experience during the first few weeks of life was less important [47]. Therefore, the layer chick’s early preference for substrate does not appear to influence their choice in the later life [47].

Studies have shown that ectoparasites significantly increase the frequency of preening, leading to feather disorders, skin damage, anemia, slow growth rate, and a decrease in egg production, as well as making the hosts vulnerable to pecking [56]. Providing pullets with sand, peat, and straw for dust bathing at an early age stimulates activity, thereby promoting feather, leg, and breast condition, as well as preventing parasites such as mites and lice and reducing body fat accumulation [48,49,57]. Sand is a common litter material that easily penetrates layer chicks’ and pullets’ feathers and helps remove parasites and sebum compared to other litter materials such as wood shavings, rice hulls, oat husks, straw pellets, and paper scraps [44,58,59]. In addition, the provision of peat moss has been shown to be beneficial for chicks and pullets compared to others because it absorbs sebum easily [60,61]. However, Dixon et al. (2010) observed that sand bath substrates do not reduce feather pecking when provided as a sole manipulable material in the chicken coop [50]. Consistently, previous studies reported that if only dust-bathing materials are provided and foraging materials are absent, pecking behavior is not reduced.

On the contrary, pullets exposed to straw materials such as rice hulls and oat husks reduce pecking behavior and increase foraging behavior [19,50,62]. Furthermore, a previous study indicated that dust-bathing substrates might increase feather-pecking injuries compared with foraging materials [19]. Therefore, focusing on the use of suitable foraging substrate is important. This was confirmed by a previous study on the Y-maze test after feed deprivation. The results showed that pullets chose food more frequently and faster than sand, suggesting that foraging behavior is instinctive [63]. This suggests that materials that promote foraging can effectively reduce or delay pecking addiction.

Manipulable materials can reduce behavioral disorders; therefore, several studies have compared the applicability of different litter substrates, especially the form of the substrates. For instance, Huber-Eicher reported that long straw prevents severe feather pecking more than short straw and polystyrene blocks [45]. Similarly, compared with 19-week-old white-shell layers with access to long-cut straw, layers fed pellet diet without access to straw showed severe feather damage, whereas those fed powder diet without access to straw were in the middle [51]. In addition, supplementing forage or litter materials with grain or feed can increase the population of mites in the feathers, thereby endangering the welfare of birds [54,64]. Furthermore, a study showed that the provision of hay to adult layers reduced mild pecking either because the hay was not sufficiently attractive to the layers or due to late provision [52]. In addition, silage corn straw, pea–barley silage, or carrots as additional enrichment materials have significantly reduced feather pecking, feather damage, and mortality among brown-shell layers [65]. However, farms should use silage and other perishables prudently because of potential nutritional imbalances, and health and safety concerns [65]. Therefore, while it is important to completely eradicate feather pecking, it cannot be achieved with only the provision of litter material [66], regardless of the quality of litter materials and the supplementation time.

In cage rearing, laying hens within five weeks old are constantly kept in metal cages without litter. Data collected from five pullet farms in Norway showed that the group of layers provided with chick paper from day one showed less feather damage and fear at the age of 30 weeks than those without access to chick paper [12,46]. Partial or complete removal of chick paper from the cages without other enrichment materials can result in a decrease of foraging behavior and increase in the frequency of severe feather pecking [8]. Chick paper supply promotes foraging, reduces feather pecking, and protects the chicks’ feet by providing good support. Moreover, it also provides the required fecal contact for a successful coccidiosis vaccination and reduces anxiety reactions among the layers [67].

Therefore, early and continuous provision with large volumes of dry, clean and fluffy litter from the first few days of the layers’ life is required to positively impact the welfare of chicks and pullets. However, some litter materials such as long straw and chick paper are suitable for foraging and are better than dust bathing materials or other new materials for layer chicks and pullets [8].

### 3.2. Stone or String for Pecking

Pecking stone and string are convenient and effective materials for enriching the environment for layer chicks and pullets. These enrichment materials are specifically suitable for cages with wired floors. Their impact on layer chicks and pullets were reported in previous studies and are summarized in Table 2.

The provision of natural shelters equipped with porous carbon residues, pecking stones, or pecking blocks can attract the attention of cage-free pullets and shorten their beaks by increasing the abrasion of keratin from the upper beak. They therefore effectively reduce severe feather pecking, aggressive pecking, plasma corticosterone level, and fear and mortality among the birds [69,77,78]. In addition, caged pullets use pecking stones or other pecking materials more frequently in the evening, as the birds tend to forage more at dusk [70]. However, in commercial pullets’ houses, the provision of pecking stones was not always effective, and different study sites of the same experiment sometimes obtained opposite results, which could be related to other factors or environmental conditions [71]. For example, a study showed that the occurrence of feather damage during the rearing period is significantly associated with the increase in the temperature of the housing system and the chronological age of pullets [76]. Moreover, the efficiency of the pecking stones is not consistent among different breeds of birds. For instance, a study observed that Lohmann selected Leghorn pullets provided with pecking stones during the rearing and laying periods showed higher toe injuries and less plumage damage, whereas Bovans Brown recorded more serious plumage damage and skin injuries compared to the hens in the control. The hens in the control group are those that did not receive additional enrichment materials such as pecking stones and alfalfa bales [72]. Furthermore, different strains of hens prefer specific pecking materials. Because of the diverging characteristics between strains, future practical recommendations for laying hen husbandry should be strain-specific [79].

Commonly, chicks and pullets raised in metal cages make intensive use of enrichment materials such as textile strings, especially when they are provided with such materials at a very young age [73]. Chicks prefer white or yellow strings over red, green, or blue ones; and the length and width of the bunches of string make them non-detectable for pecking [74]. A study on white Leghorn pullets showed that the earlier and higher the frequency of the supply of the pecking string, the lower the frequency of feather pecking. In addition, it was revealed that at 35 weeks old, the feather condition of Roman layers with access to pecking strings from 16 weeks old was significantly better than those without access to pecking strings [75]. These studies focus on improving the feather condition of the layers. Conversely, Schreiter (2019) reported no consistent effect on reducing feather and skin damage using enriched materials such as alfalfa bales, pecking stones, grain plus litter, and pecking stones plus grain [8]. In the study conducted by Hartcher et al. (2015), enrichment was provided from 12 days of age in 3 forms, the first being the provision of pecking string devices. The second form of enrichment was the inclusion of whole oats to encourage foraging and food-searching behavior in the litter. The final form of enrichment was the provision of deeper litter’. Meanwhile, beak trimming was performed at 1 d. The authors found that there was no effect of enrichment on plumage damage; however, beak trimming appeared to be effective in reducing plumage damage in ISA brown hens at week 43 weeks old. Thus, beak trimming may be an alternative practice to reduce severe feather pecking when enrichment does not redirect the behavior [40].

In general, although there are inconsistencies in the effects of manipulating the enrichment materials, the simultaneous provision of a variety of abundant and suitable enrichment materials is effective in preventing competition-induced aggregation and asphyxia. For instance, the provision of appropriate foraging materials such as long straw or chopped straw shows better enrichment effects than dust bath materials, for example, sand and pecking stones/strings or novel materials such as polystyrene block. In addition, feeding pullets and laying hens with mash instead of pelleted feed can alleviate feather damage [8,40].

## 4. Effects of Structural Equipment

Osteoporosis usually occurs among cage-laying hens during the late laying period. Skeletal muscle development of pullets improves when pullets are reared in aviaries compared to conventional cages due to the increased locomotion of pullets. Therefore, researchers are currently focusing on optimizing the environmental management during the rearing period of birds [80]. Studies have shown that pullets provided with suitable perches such as swing rod or A-type frame structure, elevated platform or aviary with more flight opportunities [81], and enriched colony cage [82] show improved muscle deposition and bone strength [83,84,85], reduced feather pecking, vent pecking and floor eggs [86,87,88], decreased abdominal fat weight [89], and alleviated stress response. Examples of stress indicators include serum catecholamine, corticosterone, serotonin, and tryptophan and agonistic behavior [90]. Notably, suitable structural equipment helps distribute birds more evenly, thereby alleviating potential local problems associated with high stocking densities. Furthermore, early exposure to varied environmental stimuli may reduce fearfulness [16]. The positive correlations between fearfulness and feather pecking have been reported [17]. Therefore, varied environmental stimulation during an early age may contribute to better welfare. In addition, early rearing in a complex environment could increase the chances for the birds to develop skills of navigation [18]. Table 3 shows the summary of the equipment used for locomotion.

### 4.1. Perches

Chickens are highly interested in perches, especially the highest perches. This exhibits highly conserved anti-predator behavior in chickens despite many generations of domestication [98,99]. Studies have indicated that chicks start perching at the lowest level of the cage and closest against the cage wall [39]. However, at later age, the chicks prefer the highest perches [100]. Generally, the provision of enough perches during the rearing period significantly increases the birds’ perching time [99,101]. The importance of early experience with perches has been outlined in several previous studies. A study shows that Lohmann white laying hens without prior perching experience showed increasing use of perches over time. Thus, it takes up to five to seven weeks of perch exposure for young hens to show consistent perching behaviors in an enriched colony setting. The authors found that laying hens spent about 10% of daytime on the perches, and over 75% of hens perched at night after approaching consistent perching behaviors [92].

Furthermore, the physical process of jumping up onto perches is thought to be helpful for chicks and pullets to develop functional experience in locomotion, as they have to learn to move in a multi-dimensional space. Thus, the provision of perches during the rearing period improves the bone mineral contents and bone strength of the hens that are kept in conventional cages. This is important because poor bone strength may negatively impact the occurrence of keel bone damage [102]. Notably, experience in perching helps chicks to develop perceptual recognition of perches as resting or escape routes [103]. A study on 64 Swiss flocks given free access to perches during the rearing period observed less feather pecking [86]. However, impaired perching development may lead to a high frequency of collisions [83,93], inducing keel fracture, broken hind claw bones, poor breast and tail feather scores, dirty floor-eggs, and poor feed efficiency [84]. The frequency of the birds piling up into heaps increases in the perching process due to stress, which may lead to smothering [88]. Therefore, providing chicks and pullets with perches proceeded by standardized feeding management throughout the rearing period is necessary.

Roman white layers were found to have no preference for circular or hexagonal perches, despite previous exposure to perches. Moreover, the minimum space between parallel perches is 30 cm (25 cm is also feasible for limited space) [104]. In addition, cooled perches were tested during the Indiana summer on White Leghorn pullets from 16 weeks old, and the results indicated that cooled perch pullets showed a lower heterophil-to-lymphocyte (H/L) ratio than both air perch pullets and no-perch pullets at 27.6 and 32 weeks old [91], which suggested that pullets were able to cope with acute heat stress more effectively.

In conclusion, perches allow pullets to express their natural habitat instincts, and have certain effects on improving musculoskeletal performance, reducing fear, stress, and abdominal fat content. Therefore, perches are widely used during the rearing and laying periods, both in cage-free and colony cage systems. It is believed that in the future, perches will provide better welfare to the pullets in terms of shape, materials, arrangement, and comfort.

### 4.2. Ramps and Platforms

Platforms, usually in the form of plastic, are slatted areas that can be accessed via ramps. Ramps and platforms are other options that provide better locomotion for birds. Ramps are effective in improving early adaptation and later navigation capacity of the chicks in multi-storied perch systems. Generally, pullets prefer using a grid ramp when moving from the ground to the shelves, as they are less likely to slip or crash [105]; on the other hand, the stepped ramps provide a better resting place for the birds because the surface of the steps is leveled [95]. In larger aviaries, the elevated platforms act as shakers and thereby help the pullets to exercise their leg muscles as they move to and from the platform. Due to the additional possibilities for locomotion, birds with access to the platforms have better skeletal development, leg health, and gait scores [106]. Moreover, the hanging platforms with an incline of 40 degrees or less are the safest for pullets of all ages and breeds. However, as the slope becomes steeper, they need to perform more wing running and aerial movements such as jumping and short flights. Younger chicks are better at wing running than older ones, and white-feathered breeds are better than brown-feathered breeds [107]. Additionally, the enrichment equipment must be easily removable during bird catching and coop cleaning.

In summary, platforms and ramps can effectively promote locomotion and avoid disturbances when the chicks and pullets are resting. The opportunity for locomotion is necessary for both skeletal and behavioral development of chicks and pullets. However, further studies are required to determine the impact of such resting places on behavior and welfare.

### 4.3. Aviaries

The spatial navigation skills and physical fitness of pullets are closely related to the breed, age, and developmental stages [94]. For example, pullets reared in aviaries had better working memory and greater spatial navigation skills and showed less fear during the novel object tests compared with those raised in barren cages [108].

The diversity of aviaries leads pullets to make efficient use of higher parts in the aviary during the laying stage and engage in long-distance flights and jumps, which helps to reduce mortality and fosters a preference for laying in nest boxes [109]. In addition, some studies focused on the importance of experience in an aviary [110,111]. For instance, a previous study observed a decrease in feather pecking, associated with fewer red mite infestations in the laying hens raised in aviaries [112]. Furthermore, in the aviary systems, pullets and hens coped well with stress and novel materials. In contrast, fearful pullets are likely to peck and fight as adults, especially in a cage-free system [113].

Existing studies illustrated that the experience of rearing in the aviary system also exerts beneficial effects on the development of the musculoskeletal function. Another study revealed that cortical bone density of pullets raised for six weeks in the aviary equipped with slopes and elevated platforms, which encompasses features such as bone structure and the strength and hardness index of the tibia and humerus, were higher than those raised in a conventional cage for 12 weeks [81]. Similarly, at 16 weeks old, pullets with access to aviaries have heavier pectoral muscles, larger keel, higher bone density, and stronger tibia, humerus, and radius than those reared in the cage [106]. Crucially, such musculoskeletal characteristics persist until the end of the laying period, even when the hens are subsequently housed in narrower cages, suggesting that skeletal development during the rearing period affects the bone health of hens and reduces the incidence of keel fractures at the end of the laying period [82,96,106]. Furthermore, pullets exposed to the aviary at 25 weeks of age can easily adapt to this system, but it may require a longer time to adapt compared to those exposed at 17 weeks of age [114].

There are considerable differences in rearing aviary design. A recent study found that strain, as well as differences in rearing aviary design, can affect the types of locomotion that growing pullets perform, which may, in turn, impact their skeletal development [115]. Thus, it is recommended to raise pullets in a complex aviary system or a diverse environment during the early rearing period, regardless of the stereoscopic enrichment provided during the laying period [97].

In conclusion, providing a rich and complex environment including slopes, ramps, platforms, and aviaries to layer chicks and pullets may improve their musculoskeletal properties, spatial navigation ability, foot pad and feather condition as well as reduce fear among the birds.

## 5. Effects of Outdoor Access

Outdoor opportunities for birds are critical during the rearing period because early experience with access to going outdoor influences the use of outdoor areas during the adult stage. Data from farms indicated that adult hens without outdoor access in the early stage showed a lower tendency in using outside ranges at any time, whereas many adult hens with outdoor experiences continuously preferred going outside [116]. Table 4 presents the effects of outdoor access and relevant information.

In outdoor free-range environments, chicks and pullets have more opportunities to express their natural behaviors such as walking, running, jumping, foraging, and dust bathing, which can reduce severe feather pecking, improve feather conditions [112,117], and reduces fearful behaviors [55]. A study on the comparison between pullets with/without free range access and the results showed that birds exposed to an outdoor area emerged more quickly from a test box placed outside, and also moved further away from the box [120]. In addition, studies have shown that compared with the indoor hens, the high outdoor activity group for 5.2–9 h per day had fewer comb bruises, shorter toenails, good feather coverage, lower body weight or less body fat, heavier spleen and muscle stomach [121], and better spatial navigation skills [53]. Similarly, six-week-old chicks that spent a week outdoors were less fearful, located the feed reward quickly in the Y-maze test [118], and preferred natural light [122] compared with the controls without outdoor experience.

Studies have shown that behavioral repertoire of birds change with age, while the effects of light treatment are subtle. Some evidence was found that birds preferred either daylight or forest light to control light, suggesting that inclusion of UV contributed to the preference. Reports indicated that daylight and forest light are associated with more active behaviors, and daylight with better plumage and later start of lay. Thus, natural-like light may have beneficial effects on domestic fowl, but the differences between broad-spectrum light sources are rather small [122,123,124].

Therefore, it is important to expose indoor laying pullets to natural light during the rearing period before moving them to a free-range system to facilitate their adaptation to outdoor life. Furthermore, compared to an indoor system, outdoor exposure improves health and welfare, behavioral and physiological stimulation, and provides regular movement between different climatic environments to avoid adverse ambient conditions [119].

Interestingly, the proportion of laying hens using outdoor pastures rarely exceeds 50% of the flock at any time, sometimes even less than 10%, and the proportion of hens entering the outdoor activities decreases with increasing stocking density [125,126]. Among them, 2% of marked hens in each group never went outdoors, whereas 38~48% were always present on the pasture and used all available areas [127]. Thus, not all pullets make equal use of the outdoor range, and factors that impact the outdoor distribution of pullets need to be further identified to promote their chances for outdoor activities [128].

The flock size is one of the critical factors. For example, studies have indicated that relatively low feather-pecking damage is observed in the free-range flock with a smaller flock size at about 500 birds [129,130]. Therefore, it is necessary to control pullet flocks under a free-range system with low flock size. When outdoor humidity and weather are comfortable, the aged hens accompanied by roosters are more willing to go outdoors, especially in an outdoor area with shade or artificial shelters [126,129].

In addition, feed and breed also affect the distribution of outdoor flocks. The flocks observed outside were fed more moderate-energy diets than low-energy diets, and the Labresse hybrid pullets require more outdoor space than Ross 208 pullets [131]. These rules can help us promote more pullets being outside to avoid fighting and pecking.

In addition, adequate daily management and preparedness are recommended to strengthen the poultry industry so that it may be easier for pullets to conquer the outdoor risks such as infection, poor environmental sanitation, abrupt climate change, unbalanced diet, and the threat from natural enemies caused by the complex interaction of outdoor factors. For instance, a study showed that factors associated with a 13-day rainfall event during the late winter predisposed flocks to severe feather pecking. While multiple factors such as winter cold, muddy ranges, damp floor litter with elevated pH, among others coincided, hens are more impacted in south- than north-aspect pens [2].

In conclusion, when precautions are taken against possible adverse factors, outdoor access has positive effects on the health and welfare of hens, such as expression of abundant natural behaviors, reduction of fear and severe pecking, and improvement of feather conditions. Therefore, to promote the use of outdoor spaces by pullets, it is important to first select suitable strains, maintain appropriate population size and density, provide indoor enrichments, expose them to natural light or outdoor shelters at an early stage of rearing, and then strengthen the management of outdoor access.

## 6. Competence of Farm Staff

It is generally believed that avoiding underweight pullets, increasing uniformity, and avoiding pain and lameness among the flocks may reduce the risk of feather pecking and cannibalism. However, the beneficial effects of enrichments, such as providing litter, pecking, and exercise equipment on the farm, are highly dependent on the professionalism of the farm staff.

Problems related to damages caused by pecking are common in an intensive egg production system and usually require beak trimming to control them. However, the majority of respondents (74%) in Finnish layer farms indicated that 5–7% feather pecking or 1–2% cannibalism could be tolerated with proper flock management, especially lighting, and feeding, and pointing to the incorporation of no-beak-trimming polices into sustainable egg production systems [31]. It was reported that harmful pecking was significantly reduced in 53 treatment flocks compared with 47 control flocks [68]. It supports the fact that implementing a management package, including measures directed during the rearing period, significantly reduces the levels of injury caused by pecking during the laying phase. Another study conducted in the UK with flocks of 34 pullet had similar results [11], showing that the care given by experienced staff during rearing significantly reduced feather pecking during the laying period. In addition, a study involving 122 Canadian layer farms reported that the higher levels of feather damage in farms are closely associated with the characteristics of increased age, brownish feathers, midnight feeding and scratch area. The aim of this study was to assess the relationship between layers, housing, management, and feather damage in the alternative feeding system and to implement the plan to eliminate the traditional battery cage by 2036 [132].

In addition, the uniformity of pullet flocks, which is closely related to the management ability of the staff, also affects the mortality. Those flocks with poor uniformity during rearing have a higher mortality. For example, the mortality rate of Roman LSL hens kept in both traditional cages and enriched cages with uniformity of more than 90% at 15 weeks old is lower during the laying period than in the other groups [133]. Therefore, improving staff management through business guidance is the core and starting point for implementing various farm enrichments, and its practical effect is undisputable, as demonstrated by some training programs on raising pullets.

## 7. Conclusions and Future Prospective

In summary, the integrated application of various beneficial farm environmental enrichment methods can continuously attract birds to peck, forage, dust-bathe, and locomote. These activities, in turn, can greatly improve layer welfare and performance, such as reducing fear and pecking, alleviating feather and skin damage, improving musculoskeletal performance, decreasing abdominal fat content, and improving egg quality during the laying period.

Moreover, providing sufficient enrichments without interruption at an early stage of development is important. For example, the quality of the litter must be maintained, by keeping it dry, clean, and fluffy. Moreover, the litter suitable for foraging such as long straw is significantly better than the dust bath material or other novel items. In general, the location and optimal amount of farm environmental enrichment are important factors in avoiding competition or crowding. In addition, the promotion of the professional ability of farm workers is crucial to the successful management of farm environmental enrichments. Notably, due to the differences in feeding methods, flock density and scale, region temperature, seasonal temperature, and humidity, some enrichment may have different effects on the layer welfare. Therefore, it is recommended that the methods and results summarized in this review may have to be tailored for each farm and chicken breed with pilot studies.

Some enrichments, such as perches, straw bales, stone pecking, rope pecking, and chick papers can be used in the chick cages, as these improve the welfare of chickens. This is significant because there are many countries such as China that are highly dependent on cage rearing. Taken together, appropriate enrichment and management will improve the welfare of caged chicks, reducing their stress and mortality rate, as well as increasing early weight gain.

Therefore, through better understanding of the layer behavior and needs, we can improve the layer production, and thereby improve layer welfare and quality of poultry products.

## Figures and Tables

**Table 1:** Litter materials designed for layers use in different studies.

References	Materials	Testing Age	Strain Used	Impacts on Birds
Martin et al., 2012 [44]	Dust box with sand	1–8 weeks old	Hy-Line Brown	To suppress ectoparasites
Nørgaard-Nielsen et al., 1993 [48]	Cut straw from a basket	18–72 weeks old	White Leghorns	To reduce feather pecking significantly
Johnsen et al., 1998 [49]	Sand *, straw, wire	0–45 weeks old	Lohmann Brown and Lohmann selected Leghorn	To reduce feather pecking for both strains
Dixon et al., 2010 [50]	Forages *, novel objects, dustbaths	14 weeks old	White Leghorns	To reduce feather pecking, but provision of only one manipulable material shows no effect
Huber-Eicher et al., 1997a [19]	Sand and straw	0–7 weeks old	Laying hen	To reduce feather pecking
Huber-Eicher et al., 1998 [45]	Long-cut * or shredded strawPolystyrene blocks * or beads	1–5 weeks old	Laying hen	To increase foraging and reduce feather pecking
Aerni et al., 2000 [51]	Long-cut straw and mash or pellets	0–18 weeks old	White Lohman Selected Leghorn hybrids	To reduce feather pecking
Daigle et al., 2014 [52]	Hay bale	21–37 weeks old	White laying hens	To reduce conspecific pecking behavior
Tahamtani et al., 2016 [12]	Chick paper	0–32 weeks old	Lohmann selected Leghorn	To reduce the frequency of feather pecking and severe pecking
Brantsæter et al., 2017 [46]	Chick paper	0–5 weeks old	Lohmann selected Leghorn	To reduce fearfulness, two-fold birds approach the novel object
Nicol et al., 2001 [47]	Wood shavings	1–210 days old	Laying hens	To increase ground pecking, decrease feather pecking
Campbell et al., 2018 [53]	Wood shavings as a floor substrate	1–21 days old	Hy-Line Brown	To enhance birds’ adaptability to environmental stressors
Vezzoli et al., 2015 [54]	Grain or feed particles	0–11 weeks old	Laying hens	To increase the number of mites on feathers
Yan et al., 2020 [25]	Wood shavings and sand	1–32 days old	Female Weining chicks	To decrease fearfulness, and reduce plasma corticosterone
Bari MS et al., 2020 [55]	Novel objects	4 days to 16 weeks old	Hyline Brown layer	To increase egg shell and yolk color
De Haas EN et al., 2014 [17]	Wood shavings, alfalfa or cardboard paper	0–17 weeks old	ISA brown cross and Dekalb White cross	To reduce the risk of severe feather pecking

* These materials showed a better effect.

**Table 2:** Pecking materials designed for layers used in different studies.

References	Materials	Testing Age	Strain Used	Impacts on Birds
Lambton et al., 2013 [68]	Bespoke management package	20,30,40 weeks old	Loose-housed laying hens	To reduce injurious pecking
Zepp et al., 2018 [69]	Pecking stone, pecking block, and lucerne bale	day 1 to end the of rearing period	Lohmann Brown	To reduce the occurrence of GFP, SFP, and aggressive pecking
Moroki et al., 2016 [70]	Pecking stones	15 months old	White Leghorn	To reduce agonistic behavior
Iqbal et al., 2020 [71]	Pecking stones	16–46 weeks old	Hy-Line Brown	To reduce feather pecking and reduce the mortality
Schreiter et al., 2020 [72]	Pecking stones and alfalfa bales	1–18 weeks old	Lohmann Selected Leghorn	To reduce plumage damage but toe injuries more serious
			Bovans Brown	Severer plumage damage and skin injuries
Jones et al., 1999 [73]	Bunches of string *, baubles or leg bands	2–11 days old	ISA Brown	To help express natural behavior
Jones et al., 2000 [74]	Bunch of strings *, chains or beads	1–5 days old	Lohmann Brown	To reduce aggressive pecking
McAdie et al., 2005 [75]	White string device	1–57 days old	White Leghorn	To decrease feather pecking
Liebers et al., 2019 [76]	Pecking stones, pecking blocks, and lucerne bales	1–116 days old	Lohmann Brown hybrid	To increase plumage quality significantly

* These materials showed a better effect.

**Table 3:** Furnishings used for locomotion in various studies on layers.

References	Materials	Testing Age	Strain Used	Impacts on Birds
Hester et al., 2013ab [83,84]	Perches	0–71 weeks old	White Leghorns	Keel fracture, broken hind claw bones, poor breast and tail feather scores, dirty floor-eggs, and poor feed efficiency due to high frequency of collisions
BRAKE et al., 1994 [88]	Perches	0–20 weeks old	Arbor Acres breeder	To increase the frequency of birds piled up into heaps due to the panic, increase the rate of smothering
Enneking et al., 2012 [85]	Perches	0–17 weeks old	White Leghorns	To stimulate leg muscle deposition, increase the mineral content of certain bones
Yan et al., 2014 [90]	Perches	0–71 weeks old	White Leghorn	To promote skeletal development and reduce stress response
Strong et al., 2015 [91]	thermally cooled perches	16 weeks old	White Leghorn	To improve immunity and resist acute heat stress
Liu et al., 2018 [92]	Round and hexagon perches	17 weeks old	Lohmann white	To help to express natural behaviors
Baker et al., 2020 [93]	Perches	19 weeks old	Hyline W36	To increase keel bone damage
Norman et al., 2019 [94]	Frame perches, platform and ramp	1–29 days old	British Black Tail	Better spatial navigational abilities, need less time to complete the detour test
Pettersson et al., 2017 [95]	Grid ramp	3–8 weeks old	British Blacktail	To improve athlete function
Casey-Trott et al., 2017a [96]	Aviary rearing system	16 weeks old	Leghorn-Lite	To increase muscle deposition and improve bone growth: bone density, cross-sectional area, bone mineral content
Casey-Trott et al., 2017bc [81,82]	Aviary rearing system	0–16 weeks old	Lohmann selected Leghorn-Lite	To reduce the prevalence of keel-bone damage
Norman et al., 2018 [97]	Ramps	8 weeks old	British Black Tail	To reduce mobility and increase strength and cognitive ability

**Table 4:** Effects of outdoor access on chickens in different studies.

References	Materials	Testing Age	Strain Used	Impacts on Birds
Lambton et al., 2010 [117]	Mashed feed and increased range use	0–40 weeks old	Laying hens	To reduce severe feather pecking
Krause et al., 2006 [118]	Outdoor access	6 weeks old	Laying hens	Better learning and exploratory behavior
Bari et al., 2021 [55]	Free range and outdoor access	16–69 weeks old	Hy-Line Brown^®^	Less fearfulness
Rehman et al., 2018 [119]	Free range	9–18 weeks old	Laying hens	To reduce feather pecking
Campbell et al., 2018 [53]	Outdoor access	22–36 weeks old	ISA Brown	To improve spatial abilities, reduce the time to complete the T-maze test
Cronin et al., 2018 [2]	Outdoor access	6–34 weeks old	ISA Brown	To increase the pecking death rate after consecutive days of rainfall
Grigor et al., 1995 [120]	Outdoor access	12–20 weeks old	Laying hens	To increase birds’ readiness to use outdoor areas and reduce fearfulness

## Data Availability

Not applicable.

## References

[1] Vestergaard K.S., Skadhauge E., Lawson L.G. (1997). The Stress of Not Being Able to Perform Dustbathing in Laying Hens. Physiol. Behav..

[2] Cronin G.M., Hopcroft R.L., Groves P.J., Hall E.J.S., Phalen D.N., Hemsworth P.H. (2018). Why did severe feather pecking and cannibalism outbreaks occur? An unintended case study while investigating the effects of forage and stress on pullets during rearing. Poult. Sci..

[3] Dawkins R. (2015). The ontogeny of a pecking preference in domestic chicks. Z. Tierpsychol..

[4] Workman L., Andrew R.J. (1989). Simultaneous changes in behaviour and in lateralization during the development of male and female domestic chicks. Anim. Behav..

[5] Janczak A.M., Riber A.B. (2015). Review of rearing-related factors affecting the welfare of laying hens. Poult. Sci..

[6] Heerkens J.L.T., Delezie E., Ampe B., Rodenburg T.B., Tuyttens F.A.M. (2016). Ramps and hybrid effects on keel bone and foot pad disorders in modified aviaries for laying hens. Poult. Sci..

[7] Sonkamble V.V., Srivastava A.K., Pawar M.M., Chauhan H.D., Ankuya K.J., Jain A.K. (2020). Effect of Cage or Deep Litter Housing on Production Performance of White Leghorn Chickens. J. Anim. Res..

[8] Schreiter R., Damme K., von Borell E., Vogt I., Klunker M., Freick M. (2019). Effects of litter and additional enrichment elements on the occurrence of feather pecking in pullets and laying hens—A focused review. Vet. Med. Sci..

[9] Huber-Eicher B., Sebö F. (2001). Reducing feather pecking when raising laying hen chicks in aviary systems. Appl. Anim. Behav. Sci..

[10] Chow A., Hogan J.A. (2005). The development of feather pecking in Burmese red junglefowl: The influence of early experience with exploratory-rich environments. Appl. Anim. Behav. Sci..

[11] Gilani A.-M., Nicol C.J., Knowles T.G. (2013). The effect of rearing environment on feather pecking in young and adult laying hens. Appl. Anim. Behav. Sci..

[12] Tahamtani F.M., Brantsaeter M., Nordgreen J., Sandberg E., Hansen T.B., Nodtvedt A., Rodenburg T.B., Moe R.O., Janczak A.M. (2016). Effects of litter provision during early rearing and environmental enrichment during the production phase on feather pecking and feather damage in laying hens. Poult. Sci..

[13] Bari M.S., Cohen-Barnhouse A.M., Campbell D.L.M. (2020). Early rearing enrichments influenced nest use and egg quality in free-range laying hens. Animal.

[14] Aerni V., Brinkhof M.W.G., Wechsler B., Oester H., Fröhlich E. (2019). Productivity and mortality of laying hens in aviaries: A systematic review. World’s Poult. Sci. J..

[15] Riber A.B., Guzman D.A. (2016). Effects of Dark Brooders on Behavior and Fearfulness in Layers. Animals.

[16] Reed H.J., Wilkins L.J., Austin S.D., Gregory N.G. (1993). The effect of environmental enrichment during rearing on fear reactions and depopulation trauma in adult caged hens. Appl. Anim. Behav. Sci..

[17] De Haas E.N., Bolhuis J.E., de Jong I.C., Kemp B., Janczak A.M., Rodenburg T.B. (2014). Predicting feather damage in laying hens during the laying period. Is it the past or is it the present?. Appl. Anim. Behav. Sci..

[18] Gunnarsson S., Yngvesson J., Keeling L.J., Forkman B. (2000). Rearing without early access to perches impairs the spatial skills of laying hens. Appl. Anim. Behav. Sci..

[19] Huber-eicher B., Wechsler B. (1997). Feather pecking in domestic chicks: Its relation to dustbathing and foraging. Anim. Behav..

[20] De Haas E.N., Bolhuis J.E., Kemp B., Groothuis T.G., Rodenburg T.B. (2014). Parents and early life environment affect behavioral development of laying hen chickens. PLoS ONE.

[21] Kelly J.R., Borre Y., O’Brien C., Patterson E., El Aidy S., Deane J., Kennedy P.J., Beers S., Scott K., Moloney G. (2016). Transferring the blues: Depression-associated gut microbiota induces neurobehavioural changes in the rat. J. Psychiatr. Res..

[22] Hao-Ming X., Hong-Li H., You-Lian Z., Hai-Lan Z., Xu J., Di-Wen S., Yan-Di L., Yong-Jian Z., Yu-Qiang N. (2021). Fecal Microbiota Transplantation: A New Therapeutic Attempt from the Gut to the Brain. Gastroenterol. Res. Pract..

[23] Maiuolo J., Gliozzi M., Musolino V., Carresi C., Scarano F., Nucera S., Scicchitano M., Oppedisano F., Bosco F., Ruga S. (2021). The Contribution of Gut Microbiota–Brain Axis in the Development of Brain Disorders. Front. Neurosci..

[24] Jeyaraj S.E., Sivasangari K., García-Colunga J., Rajan K.E. (2021). Environmental enrichment enhances sociability by regulating glutamate signaling pathway through GR by epigenetic mechanisms in amygdala of Indian field mice *Mus booduga*. Gen. Comp. Endocrinol..

[25] Yan C., Hartcher K., Liu W., Xiao J., Xiang H., Wang J., Liu H., Zhang H., Liu J., Chen S. (2020). Adaptive response to a future life challenge: Consequences of early-life environmental complexity in dual-purpose chicks. J. Anim. Sci..

[26] Vasdal G., Vas J., Newberry R.C., Moe R.O. (2019). Effects of environmental enrichment on activity and lameness in commercial broiler production. J. Appl. Anim. Welf. Sci..

[27] Henriksen R., Groothuis T.G., Rettenbacher S. (2011). Elevated plasma corticosterone decreases yolk testosterone and progesterone in chickens: Linking maternal stress and hormone-mediated maternal effects. PLoS ONE.

[28] Henriksen R., Rettenbacher S., Ton G.G.G. (2013). Maternal corticosterone elevation during egg formation in chickens (*Gallus gallus domesticus*) influences offspring traits, partly via prenatal undernutrition. Gen. Comp. Endocrinol..

[29] Ross M., Rausch Q., Vandenberg B., Mason G. (2020). Hens with benefits: Can environmental enrichment make chickens more resilient to stress?. Physiol. Behav..

[30] Edmond A., King L.A., Solomon S.E., Bain M.M. (2005). Effect of environmental enrichment during the rearing phase on subsequent eggshell quality in broiler breeders. Br. Poult. Sci..

[31] Kaukonen E., Valros A. (2019). Feather Pecking and Cannibalism in Non-Beak-Trimmed Laying Hen Flocks—Farmers’ Perspectives. Animals.

[32] Vizzier Thaxton Y., Christensen K.D., Mench J.A., Rumley E.R., Daugherty C., Feinberg B., Parker M., Siegel P., Scanes C.G. (2016). Symposium: Animal welfare challenges for today and tomorrow. Poult. Sci..

[33] Riedstra B., Groothuis T.G.G. (2002). Early feather pecking as a form of social exploration: The effect of group stability on feather pecking and tonic immobility in domestic chicks. Appl. Anim. Behav. Sci..

[34] Rodenburg T.B., van Hierden Y.M., Buitenhuis A.J., Riedstra B., Koene P., Korte S.M., van der Poel J.J., Groothuis T.G.G., Blokhuis H.J. (2004). Feather pecking in laying hens: New insights and directions for research?. Appl. Anim. Behav. Sci..

[35] Newberry R.C., Keeling L.J., Estevez I., Bilčík B. (2007). Behaviour when young as a predictor of severe feather pecking in adult laying hens: The redirected foraging hypothesis revisited. Appl. Anim. Behav. Sci..

[36] Nicol C.J., Gregory N.G., Knowles T.G., Parkman I.D., Wilkins L.J. (1999). Differential effects of increased stocking density, mediated by increased flock size, on feather pecking and aggression in laying hens. Appl. Anim. Behav. Sci..

[37] Dixon L.M. (2009). An Investigation into the Motivation behind the Abnormal Behaviour of Feather Pecking in Laying Hens. Ph.D. Thesis.

[38] Milou J.A., Sander P., van der Franz Josef S., Rebecca E.N.E. (2012). The effect of maternal care and infrared beak trimming on development, performance and behavior of Silver Nick hens. Appl. Anim. Behav. Sci..

[39] Habinski A.M., Caston L.J., Casey-Trott T.M., Hunniford M.E., Widowski T.M. (2017). Development of perching behavior in 3 strains of pullets reared in furnished cages. Poult. Sci..

[40] Hartcher K.M., Tran K.T., Wilkinson S.J., Hemsworth P.H., Thomson P.C., Cronin G.M. (2015). The effects of environmental enrichment and beak-trimming during the rearing period on subsequent feather damage due to feather-pecking in laying hens. Poult. Sci..

[41] Rodenburg T.B., Komen H., Ellen E.D., Uitdehaag K.A., van Arendonk J.A.M. (2008). Selection method and early-life history affect behavioural development, feather pecking and cannibalism in laying hens: A review. Appl. Anim. Behav. Sci..

[42] Bilčík B., Keeling L.J. (2000). Relationship between feather pecking and ground pecking in laying hens and the effect of group size. Appl. Anim. Behav. Sci..

[43] Cloutier S., Newberry R.C. (2002). A note on aggression and cannibalism in laying hens following re-housing and re-grouping. Appl. Anim. Behav. Sci..

[44] Martin C.D., Mullens B. (2012). Housing and dustbathing effects on northern fowl mites (*Ornithonyssus sylviarum*) and chicken body lice (*Menacanthus stramineus*) on hens. Med. Vet. Entomol..

[45] Huber-eicher B., Wechsler B. (1998). The effect of quality and availability of foraging materials on feather pecking in laying hen chicks. Anim. Behav..

[46] Brantsæter M., Tahamtani F.M., Nordgreen J., Sandberg E., Hansen T.B., Rodenburg T.B., Moe R.O., Janczak A.M. (2017). Access to litter during rearing and environmental enrichment during production reduce fearfulness in adult laying hens. Appl. Anim. Behav. Sci..

[47] Nicol C.J., Lindberg A.C., Phillips A.J., Pope S.J., Wilkins L.J., Green L.E. (2001). Influence of prior exposure to wood shavings on feather pecking, dustbathing and foraging in adult laying hens. Appl. Anim. Behav. Sci..

[48] Nørgaard-Nielsen G., Vestergaard K., Simonsen H.B. (1993). Effects of rearing experience and stimulus enrichment on feather damage in laying hens. Appl. Anim. Behav. Sci..

[49] Johnsen P., Vestergaard K., NRgaard-Nielsen G. (1998). Influence of early rearing conditions on the development of feather pecking and cannibalism in domestic fowl. Appl. Anim. Behav. Sci..

[50] Dixon L.M., Duncan I.J.H., Mason G.J. (2010). The effects of four types of enrichment on feather-pecking behaviour in laying hens housed in barren environments. Anim. Welf..

[51] Aerni V., El-Lethey H., Wechsler B. (2000). Effect of foraging material and food form on feather pecking in laying hens. Br. Poult. Sci..

[52] Daigle C.L., Bolhuis J.E., Swanson J.C., Siegford J.M., Rodenburg T.B. (2014). Use of dynamic and rewarding environmental enrichment to alleviate feather pecking in non-cage laying hens. Appl. Anim. Behav. Sci..

[53] Campbell D.L.M., Talk A.C., Loh Z.A., Dyall T.R., Lee C. (2018). Spatial Cognition and Range Use in Free-Range Laying Hens. Animals.

[54] Vezzoli G., Mullens B.A., Mench J.A. (2015). Dustbathing behavior: Do ectoparasites matter?. Appl. Anim. Behav. Sci..

[55] Bari M.S., Allen S.S., Mesken J., Cohen-Barnhouse A.M., Campbell D.L.M. (2021). Relationship between Range Use and Fearfulness in Free-Range Hens from Different Rearing Enrichments. Animals.

[56] Murillo A.C., Abdoli A., Blatchford R.A., Keogh E.J., Gerry A.C. (2020). Parasitic mites alter chicken behaviour and negatively impact animal welfare. Sci. Rep..

[57] Norgaard-Nielsen G. (1997). Dustbathing and feather pecking in domestic chickens reared with and without access to sand. Appl. Anim. Behav. Sci..

[58] Shields S.J., Garner J.P., Mench J.A. (2005). Effect of Sand and Wood-Shavings Bedding on the Behavior of Broiler Chickens. Poult. Sci..

[59] Shields S.J., Garner J.P., Mench J.A. (2004). Dustbathing by broiler chickens: A comparison of preference for four different substrates. Appl. Anim. Behav. Sci..

[60] Baxter M., Bailie C.L., O’Connell N.E. (2018). An evaluation of potential dustbathing substrates for commercial broiler chickens. Animal.

[61] Olsson I.S., Keeling L.J. (2005). Why in earth? Dustbathing behaviour in jungle and domestic fowl reviewed from a Tinbergian and animal welfare perspective. Appl. Anim. Behav. Sci..

[62] Rodenburg T.B., Van Krimpen M.M., De Jong I.C., De Haas E.N., Kops M.S., Riedstra B.J., Nordquist R.E., Wagenaar J.P., Bestman M., Nicol C.J. (2013). The prevention and control of feather pecking in laying hens: Identifying the underlying principles. World’s Poult. Sci. J..

[63] Petherick J.C., Seawright E., Waddington D. (1993). Influence of motivational state on choice of food or a dustbathing/foraging substrate by domestic hens. Behav. Process..

[64] Scholz B., Urselmans S., Kjaer J.B., Schrader L. (2010). Food, wood, or plastic as substrates for dustbathing and foraging in laying hens: A preference test. Poult. Sci..

[65] Steenfeldt S., Kjaer J.B., Engberg R.M. (2007). Effect of feeding silages or carrots as supplements to laying hens on production performance, nutrient digestibility, gut structure, gut microflora and feather pecking behaviour. Br. Poult. Sci..

[66] Dixona L.M. (2008). Feather pecking behaviour and associated welfare issues in laying hens. Avian Biol. Res..

[67] Pierson F.W., Larsen C.T., Gross W.B. (1998). The effect of stress on the response of chickens to coccidiosis vaccination. Vet. Parasitol..

[68] Lambton S.L., Nicol C.J., Friel M., Main D.C.J., McKinstry J.L., Sherwin C.M., Walton J., Weeks C.A. (2013). A bespoke management package can reduce levels of injurious pecking in loose-housed laying hen flocks. Vet. Rec..

[69] Zepp M., Schwarzer A., Helmer F., Louton H., Erhard M., Schmidt P. (2018). The influence of stocking density and enrichment on the occurrence of feather pecking and aggressive pecking behavior in laying hen chicks. J. Vet. Behav..

[70] Moroki Y., Tanaka T. (2016). A pecking device as an environmental enrichment for caged laying hens. Anim. Sci. J..

[71] Iqbal Z., Drake K., Swick R.A., Taylor P.S., Perez-Maldonado R.A., Ruhnke I. (2020). Effect of pecking stones and age on feather cover, hen mortality, and performance in free-range laying hens. Poult. Sci..

[72] Schreiter R., Damme K., Freick M. (2020). Edible Environmental Enrichments in Littered Housing Systems: Do Their Effects on Integument Condition Differ Between Commercial Laying Hen Strains?. Animals.

[73] Jones R.B., Carmichael N.L. (1999). Responses of domestic chicks to selected pecking devices presented for varying durations. Appl. Anim. Behav. Sci..

[74] Jones R.B., Carmichael N.L., Rayner E. (2000). Pecking preferences and pre-dispositions in domestic chicks: Implications for the development of environmental enrichment devices. Appl. Anim. Behav. Sci..

[75] McAdie T.M., Keeling L.J., Blokhuis H.J., Jones R.B. (2005). Reduction in feather pecking and improvement of feather condition with the presentation of a string device to chickens. Appl. Anim. Behav. Sci..

[76] Liebers C.J., Schwarzer A., Erhard M., Schmidt P., Louton H. (2019). The influence of environmental enrichment and stocking density on the plumage and health conditions of laying hen pullets. Poult. Sci..

[77] Shi H., Li B., Tong Q., Zheng W. (2019). Effects of different claw-shortening devices on claw condition, fear, stress, and feather coverage of layer breeders. Poult. Sci..

[78] Iqbal Z., Drake K., Swick R.A., Perez-Maldonado R.A., Ruhnke I. (2019). Feed particle selection and nutrient intake altered by pecking stone consumption and beak length in free-range laying hens. Anim. Nutr..

[79] Milisits G., Szász S., Donkó T., Budai Z., Almási A., Pőcze O., Ujvári J., Farkas T.P., Garamvölgyi E., Horn P. (2021). Comparison of Changes in the Plumage and Body Condition, Egg Production, and Mortality of Different Non-Beak-Trimmed Pure Line Laying Hens during the Egg-Laying Period. Animals.

[80] Mench J.A. (2017). Advances in Poultry Welfare.

[81] Casey-Trott T.M., Korver D.R., Guerin M.T., Sandilands V., Torrey S., Widowski T.M. (2017). Opportunities for exercise during pullet rearing, Part I: Effect on the musculoskeletal characteristics of pullets. Poult. Sci..

[82] Casey-Trott T., Korver D., Guerin M., Sandilands V., Torrey S., Widowski T. (2017). Opportunities for exercise during pullet rearing, Part II: Long-term effects on bone characteristics of adult laying hens at the end-of-lay. Poult. Sci..

[83] Hester P.Y., Enneking S.A., Haley B.K., Cheng H.W., Einstein M.E., Rubin D.A. (2013). The effect of perch availability during pullet rearing and egg laying on musculoskeletal health of caged White Leghorn hens. Poult. Sci..

[84] Hester P.Y., Enneking S.A., Jefferson-Moore K.Y., Einstein M.E., Cheng H.W., Rubin D.A. (2013). The effect of perches in cages during pullet rearing and egg laying on hen performance, foot health, and plumage. Poult. Sci..

[85] Enneking S.A., Cheng H.W., Jefferson-Moore K.Y., Einstein M.E., Rubin D.A., Hester P.Y. (2012). Early access to perches in caged White Leghorn pullets. Poult. Sci..

[86] Huber-Eicher B., Audigé L. (1999). Analysis of risk factors for the occurrence of feather pecking in laying hen growers. Br. Poult. Sci..

[87] Gunnarsson S., Keeling L.J., Svedberg J. (1999). Effect of rearing factors on the prevalence of floor eggs, cloacal cannibalism and feather pecking in commercial flocks of loose housed laying hens. Br. Poult. Sci..

[88] Brake J., Keeley T.P., Jones R.B. (1994). Effect of age and presence of perches during rearing on tonic immobility fear reactions of broiler breeder pullets. Poult. Sci..

[89] Jiang S., Hester P.Y., Hu J.Y., Yan F.F., Dennis R.L., Cheng H.W. (2014). Effect of perches on liver health of hens. Poult. Sci..

[90] Yan F.F., Hester P.Y., Cheng H.W. (2014). The effect of perch access during pullet rearing and egg laying on physiological measures of stress in White Leghorns at 71 weeks of age. Poult. Sci..

[91] Strong R.A., Hester P.Y., Eicher S.D., Hu J., Heng-Wei C. (2015). The Effect of Cooled Perches on Immunological Parameters of Caged White Leghorn Hens during the Hot Summer Months. PLoS ONE.

[92] Liu K., Xin H., Shepherd T., Zhao Y. (2018). Perch-shape preference and perching behaviors of young laying hens. Appl. Anim. Behav. Sci..

[93] Baker S.L., Robison C.I., Karcher D.M., Toscano M.J., Makagon M.M. (2020). Keel impacts and associated behaviors in laying hens. Appl. Anim. Behav. Sci..

[94] Norman K.I., Adriaense J.E.C., Nicol C.J. (2019). The impact of early structural enrichment on spatial cognition in layer chicks. Behav. Process..

[95] Pettersson I.C., Weeks C.A., Norman K.I., Nicol C.J. (2017). The ability of laying pullets to negotiate two ramp designs as measured by bird preference and behaviour. PeerJ.

[96] Casey-Trott T., Korver D., Guerin M., Sandilands V., Torrey S., Widowski T. (2017). Rearing system affects prevalence of keel-bone damage in laying hens: A longitudinal study of four consecutive flocks. Poult. Sci..

[97] Norman K.I., Weeks C.A., Pettersson I.C., Nicol C.J. (2018). The effect of experience of ramps at rear on the subsequent ability of layer pullets to negotiate a ramp transition. Appl. Anim. Behav. Sci..

[98] Newberry R.C., Estevez I., Keeling L.J. (2001). Group size and perching behaviour in young domestic fowl. Appl. Anim. Behav. Sci..

[99] Heikkilä M., Wichman A., Gunnarsson S., Valros A. (2006). Development of perching behaviour in chicks reared in enriched environment. Appl. Anim. Behav. Sci..

[100] Riber A.B., Wichman A., Braastad B.O., Forkman B. (2007). Effects of broody hens on perch use, ground pecking, feather pecking and cannibalism in domestic fowl (*Gallus gallus domesticus*). Appl. Anim. Behav. Sci..

[101] Hester P.Y., Garner J.P., Enneking S.A., Cheng H.W., Einstein M.E. (2014). The effect of perch availability during pullet rearing and egg laying on the behavior of caged White Leghorn hens. Poult. Sci..

[102] Stratmann A., Fröhlich EK F., Gebhardt-Henrich S.G., Harlander-Matauschek A., Würbel H., Toscano M.J. (2016). Genetic selection to increase bone strength affects prevalence of keel bone damage and egg parameters in commercially housed laying hens. Poult. Sci..

[103] MC A., Ijh D. (1989). Development of perching in hens. Behav. Biol..

[104] Liu K., Xin H. (2017). Effects of horizontal distance between perches on perching behaviors of Lohmann Hens. Appl. Anim. Behav. Sci..

[105] Zheng H., Li B., Tong Q., Chen G., Li X. (2019). Modification of perchery system: Preference for ramps rather than ladders during early adaptation period for cage-reared pullets. Int. J. Agric. Biol. Eng..

[106] Regmi P., Deland T.S., Steibel J.P., Robison C.I., Haut R.C., Orth M.W., Karcher D.M. (2015). Effect of rearing environment on bone growth of pullets. Poult. Sci..

[107] LeBlanc S., Tobalske B., Quinton M., Springthorpe D., Szkotnicki B., Wuerbel H., Harlander-Matauschek A. (2016). Physical Health Problems and Environmental Challenges Influence Balancing Behaviour in Laying Hens. PLoS ONE.

[108] Tahamtani F.M., Nordgreen J., Nordquist R.E., Janczak A.M. (2015). Early Life in a Barren Environment Adversely Affects Spatial Cognition in Laying Hens (*Gallus gallus domesticus*). Front. Vet. Sci..

[109] Colson S., Arnould C., Michel V. (2008). Influence of rearing conditions of pullets on space use and performance of hens placed in aviaries at the beginning of the laying period. Appl. Anim. Behav. Sci..

[110] Brantsæter M., Tahamtani F.M., Moe R.O., Hansen T.B., Orritt R., Nicol C., Janczak A.M. (2016). Rearing Laying Hens in Aviaries Reduces Fearfulness following Transfer to Furnished Cages. Front. Vet. Sci..

[111] Tahamtani F.M., Hansen T.B., Orritt R., Nicol C., Moe R.O., Janczak A.M. (2014). Does rearing laying hens in aviaries adversely affect long-term welfare following transfer to furnished cages?. PLoS ONE.

[112] Heerkens J.L.T., Delezie E., Kempen I., Zoons J., Ampe B., Rodenburg T.B., Tuyttens F.A.M. (2015). Specific characteristics of the aviary housing system affect plumage condition, mortality and production in laying hens. Poult. Sci..

[113] Rodenburg T.B., Buitenhuis A.J., Ask B., Uitdehaag K.A., Koene P., van der Poel J.J., van Arendonk J.A.M., Bovenhuis H. (2004). Genetic and phenotypic correlations between feather pecking and open-field response in laying hens at two different ages. Behav. Genet..

[114] MacLachlan S.S., Ali A.B.A., Toscano M.J., Siegford J.M. (2019). Influence of later exposure to perches and nests on flock level distribution of hens in an aviary system during lay. Poult. Sci..

[115] Pufall A., Harlander-Matauschek A., Hunniford M., Widowski T.M. (2021). Effects of Rearing Aviary Style and Genetic Strain on the Locomotion and Musculoskeletal Characteristics of Layer Pullets. Animals.

[116] Chielo L., Pike T., Cooper J. (2016). Ranging Behaviour of Commercial Free-Range Laying Hens. Animals.

[117] Lambton S.L., Knowles T.G., Yorke C., Nicol C.J. (2010). The risk factors affecting the development of gentle and severe feather pecking in loose housed laying hens. Appl. Anim. Behav. Sci..

[118] Krause E.T., Naguib M., Trillmich F., Schrader L. (2006). The effects of short term enrichment on learning in chickens from a laying strain (*Gallus gallus domesticus*). Appl. Anim. Behav. Sci..

[119] Rehman M.S., Mahmud A., Mehmood S., Pasha T.N., Khan M.T., Hussain J. (2018). Assessing behavior in Aseel pullets under free-range, part-time free-range, and cage system during growing phase. Poult. Sci..

[120] Grigor P., Hughes B., Appleby M. (1995). Effects of regular handling and exposure to an outside area on subsequent fearfulness and dispersal in domestic hens. Appl. Anim. Behav. Sci..

[121] Md Saiful B., Laurenson Y.C.S.M., Cohen-Barnhouse A.M., Walkden-Brown S.W., Campbell D.L.M. (2020). Effects of outdoor ranging on external and internal health parameters for hens from different rearing enrichments. PeerJ.

[122] Gunnarsson S., HeikkilÃ¤ M., Hultgren J., Valros A. (2008). A note on light preference in layer pullets reared in incandescent or natural light. Appl. Anim. Behav. Sci..

[123] Osorio D., Vorobyev M., Jones C.D. (1999). Colour vision of domestic chicks. J. Exp. Biol..

[124] Wichman A., De Groot R., Håstad O., Wall H., Rubene D. (2021). Influence of Different Light Spectrums on Behaviour and Welfare in Laying Hens. Animals.

[125] Pettersson I.C., Freire R., Nicol C.J. (2016). Factors affecting ranging behaviour in commercial free-range hens. World’s Poult. Sci. J..

[126] Gilani A.M., Knowles T.G., Nicol C.J. (2014). Factors affecting ranging behaviour in young and adult laying hens. Br. Poult. Sci..

[127] Campbell D.L.M., Hinch G.N., Dyall T.R., Warin L., Little B.A., Lee C. (2017). Outdoor stocking density in free-range laying hens: Radio-frequency identification of impacts on range use. Anim. Int. J. Anim. Biosci..

[128] MS B., Csml Y., AM C.-B., SW W.-B., Dlm C. Consequences of Outdoor Ranging on External and Internal Health Parameters of Hens from Different Rearing Enrichments. Proceedings of the 31st Annual Australian Poultry Science Symposium.

[129] Hegelund L., Sørensen J.T., Kjaer J.B., Kristensen I.S. (2005). Use of the range area in organic egg production systems: Effect of climatic factors, flock size, age and artificial cover. Br. Poult. Sci..

[130] Bestman M.W.P., Wagenaar J.P. (2003). Farm level factors associated with feather pecking in organic laying hens. Livest. Prod. Sci..

[131] Nielsen B., Thomsen M., SRensen P., Young J. (2003). Feed and strain effects on the use of outdoor areas by broilers. Br. Poult. Sci..

[132] Decina C., Berke O., van Nienke S., Baes C.F., Widowski T.M., Harlander-Matauschek A. (2019). An Investigation of Associations Between Management and Feather Damage in Canadian Laying Hens Housed in Furnished Cages. Animals.

[133] Nørlem M., Petersen M.F., Mørch-Madsen A., Mikkelsen P.S., Eriksson E., Nielsen K., Rasmussen L.K., Nielsen F.B. (2014). Demonstrationsanlæg for Filtrering af Vejvand for Udledning til Ferskvandsområde: Afrapportering for Projekt Støttet af VTU—Fonden.

